# Overall and sex-specific risk factors for subjective cognitive decline: findings from the 2015–2018 Behavioral Risk Factor Surveillance System Survey

**DOI:** 10.1186/s13293-022-00425-3

**Published:** 2022-04-12

**Authors:** Karen C. Schliep, William A. Barbeau, Kristine E. Lynch, Michelle K. Sorweid, Michael W. Varner, Norman L. Foster, Fares Qeadan

**Affiliations:** 1 Division of Public Health, Department of Family and Preventive Medicine, University of Utah School of Medicine, 375 Chipeta Way, Suite A, Salt Lake City, UT 84108 USA; 2 Parkinson School of Health Sciences and Public Health, Loyola University Chicago, Maywood, Illinois USA; 3 Division of Epidemiology, Department of Internal Medicine, University of Utah, Salt Lake City, Utah USA; 4 Department of Veterans Affairs, VA Informatics and Computing Infrastructure, Salt Lake City, Utah USA; 5 Division of Gerontology, Department of Internal Medicine, University of Utah, Salt Lake City, Utah USA; 6 Division of Maternal Fetal Medicine, Department of Obstetrics and Gynecology, University of Utah, Salt Lake City, Utah USA; 7 Department of Neurology, University of Utah, Salt Lake City, Utah USA; 8 Department of Public Health Sciences, Loyola University Chicago, Parkinson School of Health Sciences and Public Health, 2160 S 1st Ave, Maywood, IL 60153 USA

**Keywords:** Subjective cognitive decline, Dementia, Cognitive dysfunction, Behavioral Risk Factor Surveillance System, Risk factors, Sex factors

## Abstract

**Background:**

Prior research indicates that at least 35% of Alzheimer’s disease and related dementia risk may be amenable to prevention. Subjective cognitive decline is often the first indication of preclinical dementia, with the risk of subsequent Alzheimer’s disease in such individuals being greater in women than men. We wished to understand how modifiable factors are associated with subjective cognitive decline, and whether differences exist by sex.

**Methods:**

Data were collected from men and women (45 years and older) who completed the U.S. Behavioral Risk Factor Surveillance System Cognitive Decline Module (2015–2018), *n* = 216,838. We calculated population-attributable fractions for subjective cognitive decline, stratified by sex, of the following factors: limited education, deafness, social isolation, depression, smoking, physical inactivity, obesity, hypertension, and diabetes. Our models were adjusted for age, race, income, employment, marital and Veteran status, and accounted for communality among risk factors.

**Results:**

The final study sample included more women (53.7%) than men, but both had a similar prevalence of subjective cognitive decline (10.6% of women versus 11.2% of men). Women and men had nearly equivalent overall population-attributable fractions to explain subjective cognitive decline (39.7% for women versus 41.3% for men). The top three contributing risk factors were social isolation, depression, and hypertension, which explained three-quarters of the overall population-attributable fraction.

**Conclusions:**

While we did not identify any differences in modifiable factors between men and women contributing to subjective cognitive decline, other factors including reproductive or endocrinological health history or biological factors that interact with sex to modify risk warrant further research.

**Supplementary Information:**

The online version contains supplementary material available at 10.1186/s13293-022-00425-3.

## Highlights

- Subjective Cognitive Decline (SCD) is one of the earliest noticeable symptoms of Alzheimer’s disease and related dementias.
- While there is no current cure for dementia, research indicates that at least 35% of dementia risk may be modifiable by decreasing exposures years or even decades before cognitive decline becomes clinically evident.
- Prior research has shown that the risk of dementia in individuals with cognitive impairment is higher in women than in men, as is the overall risk of dementia.
- Among a nationally representative population of over 200,000 adults, ages 45 years and older, SCD prevalence was 11% for both women and men.
- Women and men also had nearly equivalent overall population-attributable fractions to explain subjective cognitive decline (39.7% for women versus 41.3% for men).
- The top three contributing risk factors for both women and men were social isolation, depression, and hypertension, which explained three-quarters of the overall population-attributable fraction.

**Supplementary Information:**

The online version contains supplementary material available at 10.1186/s13293-022-00425-3.

## Background

Life expectancy in developed countries is rising, with an estimated 50% of children born in 2000 expected to live to 100 years and beyond [1]. This extended life expectancy is a public health success story; however, a consequence of this success includes higher rates of cognitive impairment and dementia, estimated to triple by 2050 [2]. Subjective cognitive decline (SCD) is the self-reported experience of worsening or more frequent confusion or memory loss. It is considered to be one of the earliest noticeable symptoms of preclinical Alzheimer’s disease (AD) [3], although as illness progresses awareness of deficits decreases [4]. Several studies have shown that individuals with SCD have increased risk of subsequent objective cognitive decline including mild cognitive impairment and AD [5–7].

While there is no current cure for dementia, research indicates that at least 35% of dementia risk may modifiable by decreasing exposures years or even decades before cognitive decline becomes clinically evident [8–10]. Key modifiable factors, identified through systematic reviews and meta-analyses, include low educational attainment, hearing loss, social isolation, depression, smoking, physical inactivity, obesity, hypertension, and diabetes [8–10]. These modifiable factors, and their relationship with cognitive impairment and dementia, vary based on sex, geography, and other factors. Since previous studies have shown that the risk of subsequent AD in individuals with cognitive impairment is higher in women than in men [11], as is the overall risk of dementia [12, 13], an improved understanding of sex-specific differences in relation to how modifiable factors may impact cognitive dysfunction risk is needed.

Identifying early risk factors for subsequent cognitive dysfunction is challenging. Research to date is largely derived from clinical rather than population-based cohorts and have focused on patients already presenting with dementia [14]. The Behavioral Risk Factor Surveillance System (BRFSS), a United States (US) nationally representative population-based survey administered by the Centers for Disease Control and Prevention, finalized its Cognitive Decline Module that captures SCD in 2015 [15]. The objective for this study was to use 2015–2018 BRFSS data to determine the population-attributable fraction (PAF) of nine known risk factors for SCD, with a focus on sex-specific disparities.

## Methods

### Study population

All men and women aged 45 years and older who completed the 2015–2018 BRFSS survey, inclusive of the Cognitive Decline Module, were considered for this study. The purpose of the BRFSS, which began in 1984, is to assess health status and health behaviors of the US population based on self-reported responses [15]. The purpose of the Cognitive Decline Module, which was offered for the first time in 2011 and finalized and approved in 2015, is to monitor SCD and its associated effect or burden in the population [16]. The Cognitive Decline Module is an optional component offered to states who can elect or decline to include it in the BRFSS survey. Data are weighted by the Centers for Disease Control and Prevention to be representative of the population on a number of demographic characteristics, including sex, age, race, education, marital status, home ownership, phone ownership (landline telephone, cellular telephone or both) and sub-state region. From 2015–2018, all 50 states plus the District of Columbia and Puerto Rico have administered the Cognitive Decline Module at least once.

### Measure for cognitive decline

SCD was determined based on a binary ‘Yes’ or ‘No’ response to the first question of the Cognitive Decline Module: ‘During the past 12 months, have you experienced confusion or memory loss that is happening more often or is getting worse?’ Individuals who responded ‘Yes’ or ‘No’ to this question were included in the analysis (*n* = 216,838), while those who answered ‘Don’t Know/Not Sure’ (*n* = 1,501) or ‘Refused’ (*n* = 437) were excluded.

### Risk factors for cognitive decline

For comparability with prior research [9], we examined nine key modifiable risk factors for dementia, which were selected from a list of risk factors provided by the United Kingdom National Institute of Health and Care Excellence and the US National Institutes of Health [17, 18]. Nine binary risk factors were derived from standard BRFSS self-reported questions and defined as ‘Yes’ or ‘No’ variables [9]. Prior research has documented the reliability and validity of the BRFSS questionnaire [19, 20]. We excluded Refused, Don’t Know, Unsure, Missing, and Unknown responses. Some risk factors were not available in every year of BRFSS from 2015–2018 (Table 1).

**Table 1:** Modifiable risk factors obtained from BRFSS, 2015–2018

Risk factor	Survey question	Coding (yes/no)	Years of available data
Subjective cognitive decline	During the past 12 months, have you experienced confusion or memory loss that is happening more often or is getting worse?	Used default	All
Limited educational attainment	What is the highest grade or year of school you have completed? [*Never attended school or only kindergarten/Grades 1–8/Grades 9–11/Grade 12 or GED/College 1–3 years (includes technical school or associate’s degree)/College* ≥ *4 years (bachelor’s degree or higher)*]	Limited educational attainment (YES) was defined as *never having attended school or only kindergarten or Grades 1–8*. All higher levels of education were classified as NO	All
Deafness	Are you deaf or do you have serious difficulty hearing? [*Yes/No*]	Used default	2016–2018
Social isolation	1. How often do you get the social and emotional support you need? [*Always/Usually/Sometimes/Rarely/Never*] 2. Arthritis limited social activities by [*A Lot/A Little/Not Limited/Don’t Have Arthritis*]	Social isolation (YES) was defined as *never or rarely receiving needed social and emotional support and/or having social activities limited a lot by arthritis*. Social isolation (NO) was defined as *always, usually, or sometimes receiving needed social and emotional support*. Respondents who did not answer question 1 or 2 or who did not answer question 1 and answered “A Little”, “Not Limited” or “Don’t Have Arthritis” were excluded	1. 2015–2017 2. 2015, 2017
Depression	Have you ever been told you have a depressive disorder including depression, major depression, dysthymia, or minor depression? [*Yes/No*]	Used default	All
Smoking	Have you smoked at least 100 cigarettes in your lifetime and are you currently smoking every day or some days? [*Yes/No*]	Used default	All
Physical inactivity	Have you engaged in physical activity or exercise during the past 30 days other than your regular job? [*Yes/No*]	Used default	All
Obesity	What is your body weight status as determined by Body Mass Index (BMI)? [*Underweight (*≤ *18.49 kg/m*^*2*^*)/Healthy (18.50–24.99)/Overweight (25.00–29.99)/Obese (*≥ *30.00)*]	Obesity (YES) was defined as a body weight of *obese*, while Obesity (NO) was defined as a body weight of *underweight, healthy, or overweight*	All
Hypertension	Have you ever been told by a doctor, nurse, or other health professional that you have high blood pressure? [*Yes/No*]	Hypertension (NO) was defined as *never having had high blood pressure*, *or only having had borderline-high blood pressure or pre-hypertension*, *or only having had high blood pressure during pregnancy*. All other cases of high blood pressure were classified as YES	All
Diabetes	Have you ever been told that you have diabetes? [*Yes/No*]	Diabetes (NO) was defined as *never having had diabetes*, *or only having had pre-diabetes or borderline diabetes*, *or only having had diabetes during pregnancy*. All other cases of diabetes were classified as YES	All

Statistical analysis		
Variable	States with available data	Sample size^a^
Subjective cognitive decline	Alabama, Alaska, Arizona, Arkansas, Delaware, District of Columbia, Georgia, Hawaii, Idaho, Illinois, Indiana, Iowa, Kentucky, Louisiana, Massachusetts, Minnesota, Mississippi, Missouri, Montana, Nevada, New Hampshire, New Jersey, New Mexico, North Carolina, North Dakota, Ohio, Oregon, Pennsylvania, Rhode Island, South Carolina, South Dakota, Tennessee, Vermont, Virginia, Washington, West Virginia, Wisconsin, Wyoming, Puerto Rico	216,838
Limited educational attainment	Alabama, Alaska, Arizona, Arkansas, Delaware, District of Columbia, Georgia, Hawaii, Idaho, Illinois, Indiana, Iowa, Kentucky, Louisiana, Massachusetts, Minnesota, Mississippi, Missouri, Montana, Nevada, New Hampshire, New Jersey, New Mexico, North Carolina, North Dakota, Ohio, Oregon, Pennsylvania, Rhode Island, South Carolina, South Dakota, Tennessee, Vermont, Virginia, Washington, West Virginia, Wisconsin, Wyoming, Puerto Rico	216,231
Deafness	Alaska, Delaware, Georgia, Hawaii, Idaho, Indiana, Kentucky, Massachusetts, Mississippi, Missouri, Montana, New Hampshire, New Jersey, New Mexico, North Carolina, Oregon, Pennsylvania, Tennessee, Vermont, Washington, Puerto Rico	100,825
Social isolation	Alabama, Arizona, Arkansas, District of Columbia, Georgia, Hawaii, Illinois, Iowa, Louisiana, Minnesota, Mississippi, Nevada, New Jersey, North Dakota, Ohio, Oregon, Rhode Island, South Carolina, South Dakota, Tennessee, Virginia, West Virginia, Wisconsin, Wyoming, Puerto Rico	Total: 28,436
		1. 18,168
		2. 131,738 (11,102 for ‘arthritis limited social activities by a lot’)
Depression	Alabama, Alaska, Arizona, Arkansas, Delaware, District of Columbia, Georgia, Hawaii, Idaho, Illinois, Indiana, Iowa, Kentucky, Louisiana, Massachusetts, Minnesota, Mississippi, Missouri, Montana, Nevada, New Hampshire, New Jersey, New Mexico, North Carolina, North Dakota, Ohio, Oregon, Pennsylvania, Rhode Island, South Carolina, South Dakota, Tennessee, Vermont, Virginia, Washington, West Virginia, Wisconsin, Wyoming, Puerto Rico	215,970
Smoking	Alabama, Alaska, Arizona, Arkansas, Delaware, District of Columbia, Georgia, Hawaii, Idaho, Illinois, Indiana, Iowa, Kentucky, Louisiana, Massachusetts, Minnesota, Mississippi, Missouri, Montana, Nevada, New Hampshire, New Jersey, New Mexico, North Carolina, North Dakota, Ohio, Oregon, Pennsylvania, Rhode Island, South Carolina, South Dakota, Tennessee, Vermont, Virginia, Washington, West Virginia, Wisconsin, Wyoming, Puerto Rico	215,536
Physical inactivity	Alabama, Alaska, Arizona, Arkansas, Delaware, District of Columbia, Georgia, Hawaii, Idaho, Illinois, Indiana, Iowa, Kentucky, Louisiana, Massachusetts, Minnesota, Mississippi, Missouri, Montana, Nevada, New Hampshire, New Jersey, New Mexico, North Carolina, North Dakota, Ohio, Oregon, Pennsylvania, Rhode Island, South Carolina, South Dakota, Tennessee, Vermont, Virginia, Washington, West Virginia, Wisconsin, Wyoming, Puerto Rico	216,395
Obesity	Alabama, Alaska, Arizona, Arkansas, Delaware, District of Columbia, Georgia, Hawaii, Idaho, Illinois, Indiana, Iowa, Kentucky, Louisiana, Massachusetts, Minnesota, Mississippi, Missouri, Montana, Nevada, New Hampshire, New Jersey, New Mexico, North Carolina, North Dakota, Ohio, Oregon, Pennsylvania, Rhode Island, South Carolina, South Dakota, Tennessee, Vermont, Virginia, Washington, West Virginia, Wisconsin, Wyoming, Puerto Rico	204,233
Hypertension	Alabama, Arizona, Arkansas, District of Columbia, Georgia, Hawaii, Illinois, Iowa, Louisiana, Minnesota, Mississippi, Nevada, New Jersey, North Dakota, Ohio, Oregon, Rhode Island, South Carolina, South Dakota, Tennessee, Virginia, West Virginia, Wisconsin, Wyoming, Puerto Rico	132,662
Diabetes	Alabama, Alaska, Arizona, Arkansas, Delaware, District of Columbia, Georgia, Hawaii, Idaho, Illinois, Indiana, Iowa, Kentucky, Louisiana, Massachusetts, Minnesota, Mississippi, Missouri, Montana, Nevada, New Hampshire, New Jersey, New Mexico, North Carolina, North Dakota, Ohio, Oregon, Pennsylvania, Rhode Island, South Carolina, South Dakota, Tennessee, Vermont, Virginia, Washington, West Virginia, Wisconsin, Wyoming, Puerto Rico	216,483

^a^Total *n* used in analysis of adults aged 45 and older from 2015 to 2018, excluding “Don’t Know” and “Refused” responses

### Statistical analysis

Due to the complex survey design of BRFSS, statistical analyses incorporated clustering (*PSU*), stratification (*STSTR*), and weighting (*LLCPWT*), following established Centers for Disease Control and Prevention methodology, which uses iterative proportional fitting weighting since 2011 [21]. All results provided in this study were weighted estimates adjusted for disproportionate non-response, non-coverage, and other sampling bias. Descriptive statistics of respondent demographics were calculated for the total population, as well as by sex. For each risk factor, relative risk (RR) and 95% confidence intervals (CI) for SCD were estimated, both unadjusted and adjusted for demographic factors including age, race, income, employment, marital status, and Veteran status. Given that age of a respondent at the time they completed the survey may act as an effect modifier between sex or modifiable risk factors and SCD, we ran our analyses stratified by age (< 65 years versus ≥ 65 years). Principal component analysis based on a weighted tetrachoric correlation matrix of the nine risk factors was performed to calculate communality of each risk factor, which refers to the total amount of shared variance among risk factors. Due to current limitations in statistical software, stratification and clustering were not incorporated in the calculations for communality. The PAF for each risk factor and all risk factors combined (overall PAF) were calculated using formulas (see Additional file 1) provided by Livingston et al. and Mukadam et al. [8, 9, 22], with weighting to account for communality, as well as adjustment for demographic factors (WaPAF). All statistical analyses were conducted using SAS 9.4 (Cary, North Carolina, USA).

## Results

### Population characteristics

The study consisted of more women (53.7%) than men, with more women than men aged 70 years or older (Table 2). Three-quarters of the study population were non-Hispanic white, 11.1% were non-Hispanic black, 8.6% were Hispanic, and 5.0% were either non-Hispanic Asian/Native Hawaiian/Pacific Islander, non-Hispanic American Indian/Alaskan Native, or non-Hispanic individuals of other or multiple races. Compared to women, men were more likely to be younger at the time of interview, married, employed, have a higher income, and have Veteran status. Women were more than twice as likely to be widowed as men. Overall, 10.9% of adults reported experiencing symptoms of SCD, with slightly more men (11.2%) compared to women (10.6%) reporting SCD. The relationship between respondent’s age and report of SCD differed between men and women. For men, a near linear relationship between age and probability of SCD was found. For women, the relationship was more “S” shaped with women ages 50–54 showing a higher probability of SCD compared to women ages 60–64 or 70–74 (see Additional file 2).

**Table 2:** Demographic characteristics of U.S. adults aged 45 years and older by sex, 2015–2018

Characteristic	Frequency^2^ (%^3^)			*p*-value^1^
	Men	Women	Total	
Sex	88,216 (46.30)	128,622 (53.70)	216,838 (100.00)	–
Age				< 0.0001
45–49	8,787 (14.21)	11,066 (12.62)	19,853 (13.36)	
50–54	11,050 (17.42)	14,662 (16.62)	25,712 (16.99)	
55–59	12,894 (15.96)	17,573 (14.76)	30,467 (15.32)	
60–64	14,285 (16.09)	19,570 (15.79)	33,855 (15.93)	
65–69	14,501 (13.00)	20,043 (12.53)	34,544 (12.75)	
70–74	10,605 (9.70)	16,215 (10.08)	26,820 (9.90)	
75–79	7,152 (6.65)	11,997 (7.92)	19,149 (7.33)	
80 or older	8,069 (6.97)	15,665 (9.68)	23,734 (8.42)	
Race/ethnicity^†^				0.028
White	69,410 (75.14)	100,092 (75.49)	169,502 (75.33)	
Black	6048 (10.86)	11,446 (11.23)	17,494 (11.06)	
Hispanic	5127 (8.65)	7974 (8.54)	13,101 (8.59)	
Asian/Native Hawaiian/Pacific Islander	2154 (2.60)	2604 (2.35)	4758 (2.46)	
American Indian/Alaskan Native	1282 (1.01)	1680 (0.89)	2962 (0.94)	
Multiracial	2101 (1.34)	2552 (1.17)	4653 (1.25)	
Other	503 (0.41)	510 (0.32)	1013 (0.36)	
Marital status				< 0.0001
Married	55,354 (65.61)	62,710 (55.53)	118,064 (60.21)	
Divorced/separated	14,862 (16.83)	24,232 (18.01)	39,094 (17.46)	
Widowed	7659 (6.87)	29,886 (18.14)	37,545 (12.92)	
Never married	8291 (8.62)	9116 (6.61)	17,407 (7.54)	
Member of an unmarried couple	1625 (2.06)	1848 (1.71)	3473 (1.87)	
Income				< 0.0001
Less than $15,000	6791 (9.50)	13,779 (12.80)	20,570 (11.21)	
$15,000 to less than $25,000	10,935 (14.94)	19,679 (18.50)	30,614 (16.78)	
$25,000 to less than $35,000	7829 (9.73)	12,211 (10.91)	20,040 (10.34)	
$35,000 to less than $50,000	11,265 (13.87)	14,972 (13.65)	26,237 (13.76)	
$50,000 or more	40,478 (51.96)	42,923 (44.14)	83,401 (47.91)	
Employment status				< 0.0001
Employed for wages	30,405 (39.56)	38,264 (34.47)	68,669 (36.82)	
Self-employed	10,197 (11.50)	7285 (5.61)	17,482 (8.34)	
Out of work	3161 (4.57)	3891 (3.77)	7052 (4.14)	
A homemaker	219 (0.31)	11,531 (10.77)	11,750 (5.93)	
A student	120 (0.11)	326 (0.31)	446 (0.22)	
Retired	36,437 (34.39)	54,246 (34.41)	90,683 (34.40)	
Unable to work	7197 (9.57)	12,365 (10.65)	19,562 (10.15)	
Veteran status				< 0.0001
Veteran	31,046 (30.04)	2701 (2.11)	33,747 (15.03)	
Not a veteran	57,006 (69.96)	125,850 (97.89)	182,856 (84.97)	
Subjective Cognitive Decline^^^				0.021
Yes	9766 (11.15)	13,239 (10.60)	23,005 (10.86)	
No	78,450 (88.85)	115,383 (89.40)	193,833 (89.14)	

Data aggregated from U.S. Behavioral Risk Factor Surveillance System, 2015–2018; estimates were weighted and adjusted for complex survey design

^1^Chi-square test measuring association between gender and demographic characteristics

^2^Unweighted frequency

^3^Weighted column or row percentage

^†^All race/ethnicity categories are non-Hispanic, unless otherwise noted

^^^Experienced confusion or memory loss during the past 12 months that is happening more often or is getting worse (‘Yes’); otherwise (‘No’)

### Relative risk for subjective cognitive decline

Among all adults, exposure to any of the nine identified risk factors was positively associated with the risk of SCD in the unadjusted models (Table 3) and after adjusting for age, race, income, employment status, marital status, and Veteran status (Table 4). The leading risk factors included depression, social isolation, and deafness, where adults with depressive disorders were three times more likely to have SCD than adults without (adjusted risk ratio (aRR): 3.12, 95% CI 2.95, 3.29), and adults who did versus did not experience social isolation or deafness carried more than double the risk of SCD (aRR: 2.46, 95% CI 2.15, 2.77; aRR: 2.01, 95% CI 1.82, 2.19, respectively). Adults who were physically inactive (aRR: 1.32, 95% CI 1.25, 1.39), had hypertension (aRR: 1.28, 95% CI 1.20, 1.36) or diabetes (aRR: 1.28, 95% CI 1.21, 1.35), or currently smoked (aRR: 1.20, 95% CI 1.12, 1.27) carried a 20–32% increased risk for SCD compared to their non-exposed counterparts. Income was found to significantly modify the association of educational attainment, social isolation, physical inactivity, obesity, and diabetes with SCD (see Additional file 3).

**Table 3:** Unadjusted modifiable risk factors for subjective cognitive decline in U.S. adults aged 45 years and older, 2015–2018

Risk factor	RR^1^ (95% CI^2^)	Prevalence (%)	Communality^3^ (%)	PAF^4^ (%)	Weighted PAF^5^ (%)
All adults					
Limited education^a^	1.60 (1.47–1.76)	4.78	66.19	2.81	0.85
Deafness^b^	2.60 (2.41–2.80)	9.66	39.81	13.38	4.05
Social isolation^c^	3.39 (3.10–3.71)	52.38	70.97	55.60	16.82
Depression^d^	4.17 (4.01–4.34)	18.76	56.15	37.30	11.29
Smoking^e^	1.71 (1.63–1.80)	15.46	64.26	9.90	3.00
Physical inactivity^f^	1.76 (1.68–1.83)	30.76	39.83	18.87	5.71
Obesity^g^	1.27 (1.22–1.33)	32.97	59.06	8.25	2.50
Hypertension^h^	1.62 (1.53–1.71)	50.99	57.99	23.95	7.25
Diabetes^i^	1.67 (1.59–1.75)	17.76	57.65	10.57	3.20
			Overall^6^	89.31	54.66
Women					
Limited education^a^	1.61 (1.42–1.83)	4.49	58.99	2.66	0.78
Deafness^b^	2.68 (2.37–3.02)	7.46	48.90	11.12	3.28
Social isolation^c^	3.23 (2.89–3.62)	55.70	69.20	55.41	16.31
Depression^d^	4.31 (4.07–4.55)	22.92	57.33	43.12	12.70
Smoking^e^	1.88 (1.76–2.01)	14.41	65.83	11.27	3.32
Physical inactivity^f^	1.79 (1.69–1.89)	32.25	41.35	20.23	5.96
Obesity^g^	1.40 (1.32–1.49)	32.08	64.84	11.35	3.34
Hypertension^h^	1.59 (1.48–1.70)	49.57	58.25	22.52	6.63
Diabetes^i^	1.74 (1.63–1.86)	16.73	59.35	10.98	3.23
			Overall^6^	90.51	55.55
Men					
Limited education^a^	1.60 (1.40–1.82)	5.11	26.67	2.96	1.01
Deafness^b^	2.54 (2.30–2.80)	12.22	20.30	15.82	5.38
Social isolation^c^	3.65 (3.16–4.21)	47.68	71.96	55.82	19.00
Depression^d^	4.33 (4.08–4.60)	13.94	39.75	31.72	10.80
Smoking^e^	1.54 (1.43–1.67)	16.68	50.75	8.29	2.82
Physical inactivity^f^	1.73 (1.62–1.85)	29.03	35.75	17.53	5.97
Obesity^g^	1.15 (1.08–1.23)	33.93	48.34	4.85	1.65
Hypertension^h^	1.65 (1.52–1.79)	52.63	57.98	25.55	8.70
Diabetes^i^	1.59 (1.48–1.70)	18.95	54.94	10.05	3.42
			Overall^6^	88.12	58.75

Data aggregated from U.S. Behavioral Risk Factor Surveillance System, 2015–2018; estimates were weighted and/or adjusted for complex survey design. Not all risk factors were available for every year of the survey. RR and PAF were unadjusted for demographic factors

^1^Relative risk and ^2^confidence intervals for subjective cognitive decline. ^3^Total amount of variance a risk factor shares with the other factors

^4^Population-attributable fraction; proportion of all cases of subjective cognitive decline in the population that is attributable to a risk factor

^5^PAF after accounting for communality. ^6^Combined PAF of all risk factors. ^3−6^Please see Additional file 1 for formulas

^a^Never attended school or discontinued after 8th grade

^b^Includes serious difficulty hearing

^c^Never or rarely receive needed social and emotional support and/or have social activities significantly limited by arthritis

^d^Ever told to have a depressive disorder

^e^Currently smoke every day or some days and have smoked ≥ 100 cigarettes in lifetime

^f^No physical activity or exercise during past 30 days other than regular job

^g^Body mass index (BMI) ≥ 30.00 kg/m^2^

^h^Ever told to have high blood pressure by a health professional, excluding during pregnancy or borderline high, pre-hypertension

^i^Ever told to have diabetes, excluding gestational, borderline or pre-diabetes

**Table 4:** Adjusted modifiable risk factors for subjective cognitive decline in U.S. adults aged 45 years and older, 2015–2018

Risk factor	Adj RR^1^ (95% CI^2^)	Prevalence (%)	Communality^3^ (%)	Adj PAF^4^ (%)	Weighted Adj PAF^5^ (%)
All adults					
Limited education^a^	1.12 (0.99–1.26)	4.78	66.19	0.59	0.20
Deafness^b^	2.01 (1.82–2.19)	9.66	39.81	8.87	2.96
Social isolation^c^	2.46 (2.15–2.77)	52.38	70.97	43.28	14.44
Depression^d^	3.12 (2.95–3.29)	18.76	56.15	28.47	9.50
Smoking^e^	1.20 (1.12–1.27)	15.46	64.26	2.97	0.99
Physical inactivity^f^	1.32 (1.25–1.39)	30.76	39.83	8.92	2.98
Obesity^g^	1.14 (1.08–1.19)	32.97	59.06	4.32	1.44
Hypertension^h^	1.28 (1.20–1.36)	50.99	57.99	12.57	4.20
Diabetes^i^	1.28 (1.21–1.35)	17.76	57.65	4.78	1.59
			Overall^6^	74.13	38.30
Women					
1 Limited education^a^	1.18 (0.97–1.38)	4.49	58.99	0.79	0.26
Deafness^b^	2.09 (1.79–2.38)	7.46	48.90	7.50	2.44
Social isolation^c^	2.48 (2.07–2.89)	55.70	69.20	45.21	14.72
Depression^d^	3.26 (3.01–3.50)	22.92	57.33	34.08	11.09
Smoking^e^	1.29 (1.18–1.40)	14.41	65.83	4.00	1.30
Physical inactivity^f^	1.32 (1.23–1.42)	32.25	41.35	9.45	3.07
Obesity^g^	1.14 (1.06–1.22)	32.08	64.84	4.37	1.42
Hypertension^h^	1.26 (1.15–1.37)	49.57	58.25	11.33	3.69
Diabetes^i^	1.32 (1.22–1.42)	16.73	59.35	5.10	1.66
			Overall^6^	76.81	39.65
Men					
Limited education^a^	1.05 (0.87–1.24)	5.11	26.67	0.28	0.11
Deafness^b^	1.93 (1.68–2.17)	12.22	20.30	10.16	3.86
Social isolation^c^	2.47 (1.98–2.96)	47.68	71.96	41.20	15.66
Depression^d^	3.23 (2.94–3.51)	13.94	39.75	23.68	9.00
Smoking^e^	1.11 (1.01–1.22)	16.68	50.75	1.86	0.71
Physical inactivity^f^	1.32 (1.22–1.42)	29.03	35.75	8.50	3.23
Obesity^g^	1.13 (1.04–1.21)	33.93	48.34	4.14	1.58
Hypertension^h^	1.32 (1.20–1.44)	52.63	57.98	14.44	5.49
Diabetes^i^	1.24 (1.14–1.34)	18.95	54.94	4.37	1.66
			Overall^6^	71.68	41.30

Data aggregated from U.S. Behavioral Risk Factor Surveillance System, 2015–2018; estimates were weighted and/or adjusted for complex survey design. Not all risk factors were available for every year of the survey. RR and PAF were adjusted (adj) for age, race, income, employment status, marital status, and veteran status

^1^Relative risk and ^2^confidence intervals for subjective cognitive decline. ^3^Total amount of variance a risk factor shares with the other factors

^4^Population-attributable fraction; proportion of all cases of subjective cognitive decline in the population that is attributable to a risk factor

^5^PAF after accounting for communality. ^6^Combined PAF of all risk factors. ^3−6^Please see Additional file 1 for formulas

^a^Never attended school or discontinued after 8th grade

^b^Includes serious difficulty hearing

^c^Never or rarely receive needed social and emotional support and/or have social activities significantly limited by arthritis

^d^Ever told to have a depressive disorder

^e^Currently smoke every day or some days and have smoked ≥ 100 cigarettes in lifetime

^f^No physical activity or exercise during past 30 days other than regular job

^g^Body mass index (BMI) ≥ 30.00 kg/m^2^

^h^Ever told to have high blood pressure by a health professional, excluding during pregnancy or borderline high, pre-hypertension

^i^Ever told to have diabetes, excluding gestational, borderline or pre-diabetes

Across three different age cohorts (45–59; 60–74; ≥ 75), deafness, depression, and social isolation consistently showed significant risks with SCD. Notably, among those 75 or old, deafness (aRR: 2.06, 95% CI 1.64, 2.48), depression (aRR: 2.62, 95% CI 2.27, 2.98), and social isolation (aRR: 2.51, 95% CI 1.82, 3.19) were the only risk factors that were significantly associated with SCD, with a large effect size (see Additional files 4, 5, 6). Among older adults (≥ 75), associations between modifiable risk factors and SCD did not differ by sex. Among younger adults, there were differences across sex in the risk of SCD due to limited education and diabetes. In adults 45–59 years old, women with limited education had a relative risk of 1.37 (95% CI 0.97, 1.77) for SCD, whereas men with limited education had a relative risk of 3.60 (95% CI 3.11, 4.10) for SCD. Similarly, in adults 60–74 years old, women with limited education had a relative risk of 1.25 (95% CI 0.89, 1.60) for SCD, whereas men with limited education had a relative risk of 3.21 (95% CI 2.78, 3.64) for SCD. In adults 45–59 years old, women with diabetes had a relative risk of 1.22 (95% CI 1.06–1.38) for SCD, whereas men with diabetes had a relative risk of 2.48 (95% CI 1.62, 3.33) for SCD. In adults 60–74 years old, women with diabetes had a relative risk of 1.51 (95% CI 1.34, 1.68) for SCD, whereas men with diabetes had a relative risk of 2.79 (95% CI 1.99, 3.59) for SCD.

### Population-attributable fraction of risk factors

Collectively, the nine risk factors in this study were estimated to be attributed to 74.1% of all cases of SCD in the population, after adjusting for age, race, income, employment status, marital status, and Veteran status (Table 3). After accounting for the shared variance among risk factors, the hypothetical elimination of all exposures predicted a reduction in SCD by over one-third (WaPAF 38.3%). Women and men had nearly equivalent overall WaPAFs to explain SCD (39.7% for women compared to 41.3% for men). Similar findings existed among men and women of different age groups, with a WaPAF of 41.9% observed among adults < 65 and a WaPAF of 36.7% among adults ≥ 65 (see Additional files 3 and 4).

**Fig. 1:**
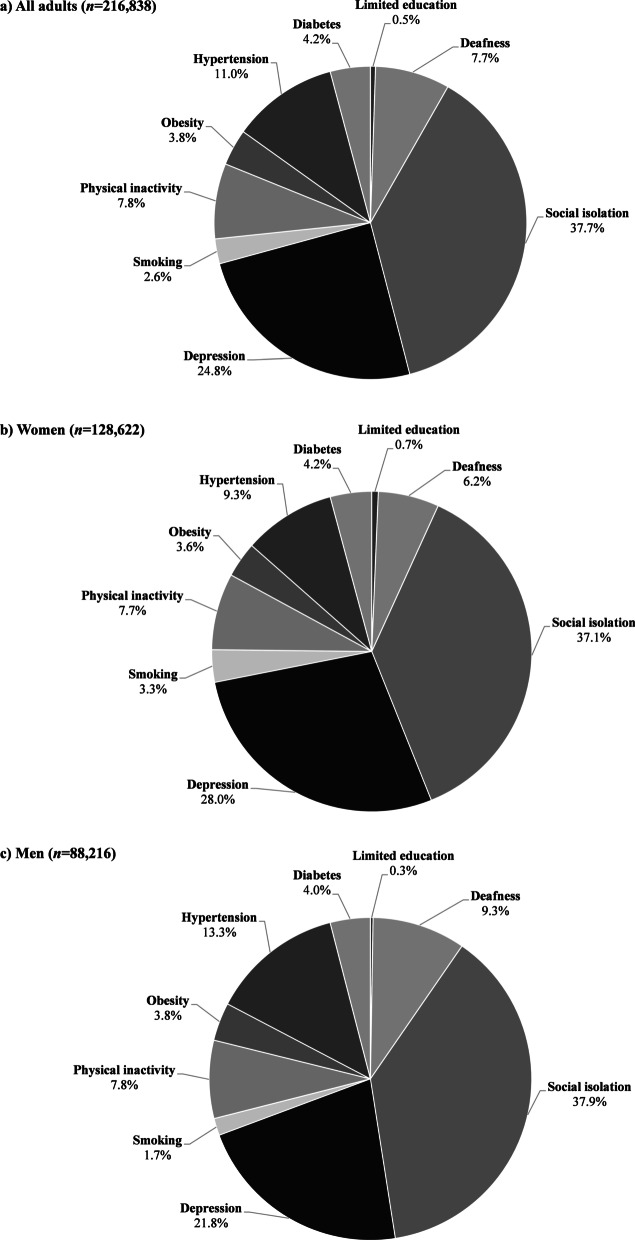
Contributions of potentially modifiable risk factors to the overall weighted adjusted population-attributable fraction for subjective cognitive decline in adults aged 45 and older (BRFSS: 2015–2018). **A** All adults (*n* = 216,838); **B** women (*n* = 128,622); **C** men (*n* = 88,216)

Nearly 38% of SCD cases in the population could be attributable to social isolation (WaPAF 14.4%), while 25% could be attributable to depression (WaPAF 9.5%) and 11% to hypertension (WaPAF 4.2%). Physical inactivity (WaPAF 3.0%), deafness (WaPAF 3.0%), diabetes (WaPAF 1.6%), obesity (WaPAF 1.4%), smoking (WaPAF 1.0%) and limited education (WaPAF 0.2%) accounted for the remaining 27% PAF for SCD (Fig. 1a). Depression, smoking, diabetes, and limited education contributed more to SCD in women than men, while social isolation, hypertension, physical inactivity, obesity, and deafness, contributed more to SCD in men than women (Table 3). However, there was little difference with regard to the magnitude of contributing factors between sexes (Fig. 1b, c). Among adults aged 75 and older, limited educational attainment (WaPAF − 0.35% for women vs WaPAF 0.085% for men) and smoking (WaPAF − 0.56% for women vs WaPAF 0.2% for men) no longer contributed to SCD risk for either women or men (see Additional file 6).

## Discussion

### Main findings

In this large representative sample of US adults aged 45 and older, we found that more than 1 in 10 survey respondents reported SCD and nearly 40% of SCD could be attributed to known modifiable factors. Overall, the top three contributing risk factors were social isolation, depression and hypertension, which contributed three-quarters of the adjusted PAF for SCD. Physical inactivity, deafness, diabetes, obesity, smoking, and limited education contributed the remaining quarter. While respondent’s age and report of SCD differed between men and women, with women reporting more cognitive complaints around the time of the menopausal transition, little difference between women and men was found for the attributable proportion of SCD that may be amenable to prevention. However, further research is warranted for exploring other modifiable factors that have recently been shown to be associated with cognitive decline, including heavy alcohol consumption, traumatic brain injury, and air pollution [8], in addition to reproductive health history [23], which may contribute to SCD risk and sex-specific SCD risk.

### Significance of results

Our study is novel in using data from a US nationally representative population-based survey to determine modifiable risk factors for subjective cognitive decline. Prior studies have used pooled estimates from meta-analyses [8–10] or smaller prospective cohorts in other countries [24, 25] to calculate PAFs for dementia. While epidemiologic evidence has shown that SCD significantly increases risk for later mild cognitive impairment and dementia, the majority of individuals with SCD (which can be caused by several medical conditions) will not go on to develop cognitive decline [26]. Current research, for which our findings may help inform, is focused on understanding what characteristics cause some individuals with SCD to go on to develop dementia compared to others who remain cognitively normal [26]. Despite different cognitive outcome assessments and study designs, we found it reassuring that our results for SCD were comparable to prior research for dementia outcomes, which demonstrate between 35 and 50% of dementia risk may be amenable to prevention [8–10, 24, 25]. Given that we had limited data for deafness, hypertension and most notably for social isolation, we advise caution in interpreting our findings as to what factors contribute the most to SCD. While our findings are in line with past research on importance of hypertension and hearing loss in relation to cognitive health, there is limited research on social isolation. Further research with gold-standard methods to capture social isolation and with complete data is needed before definitive conclusions can be made.

### Comparison with other studies

The *Lancet* Commission on Dementia Prevention, Intervention, and Care reported in 2017 that 35% of dementia cases could theoretically be preventable by eliminating the following risk factors: limited education in early life (8%), mid-life hearing loss (9%), hypertension (2%), obesity (1%), later life smoking (5%), depression (4%), physical inactivity (3%), social isolation (2%) and diabetes (1%) [9]. In a recently published 2020 report update, the Commission added three additional modifiable factors—excessive alcohol consumption (> 21 units/week), traumatic brain injury, and air pollution—which has raised the theoretical proportion of worldwide dementia that could be prevented to 40% [8].

While we determined a similar attributable fraction as the 2017 Commission (38% vs. 35%, respectively), using the same modifiable factors and methodology (WaPAF), the relative contributions of the modifiable factors differed. The Commission reported that hearing loss, limited education, and smoking contributed to over half the attributable fraction for dementia, while in the BRFSS, we found that social isolation, depression, and hypertension were responsible for nearly three-quarters of the attributable fraction for SCD.

These differences may be attributed to many factors including differences in population characteristics (e.g., clinical versus population-based samples) and the fact that the Commission assessed modifiable factors for dementia while we were assessing modifiable factors for SCD, a potential early indicator of subsequent dementia. Additionally, while we used similar methodology for calculating the PAFs for modifiable factors as the *Lancet* Commission including consideration of confounding factors for RRs and accounting for communality of risk factors [8, 9], our calculation of RR estimates for modifiable factors differed greatly from that of the Commission. They extensively reviewed the international literature conducted predominately over the past two decades on each contributing risk factor and either applied previously published pooled RRs or calculated pooled RRs based on their own meta-analysis. Furthermore, to calculate communality, they used a representative sample of over 10,000 UK community-dwelling adults [9]. For our analysis on SCD, we used a single data source within the US to calculate both our adjusted RRs and communality of risk factors within a recent specified time frame (2015–2018).

Using similar methodology to the Commission in identifying aRRs for potentially modifiable risk factors for Alzheimer’s disease via systematic reviews and meta-analyses, another prior study found an overall adjusted PAF of 54.1% for Alzheimer’s disease [10]. Given that communality was not considered, this estimate would be equivalent to the Commission’s unweighted PAF of 88.8% or our finding in the BRFSS of 74.1%. Additionally, this prior study assessed seven factors (low education, smoking, physical inactivity, depression, mid-life hypertension, diabetes and mid-life obesity) [10], so comparing impact of the additional two factors in the Commission [9] and our study—hearing loss and social isolation—is not possible. It is notable that while limited education contributed to the highest population-attributable risk in the global assessment (19.1%), and backed up by other European prospective cohort studies [23, 24], it dropped to second to last (PAR 7.3%) in the US assessment. This demonstrates the importance of calculating country-specific attributable proportions for dementia and cognitive impairment.

Our finding of an “S”-shaped relation between age and probability of SCD in women is in line with prior research [27]. Two large population-based prospective cohort studies in the US reported a 44% to 62% prevalence of subjective cognitive decline among women undergoing the menopausal transition [28, 29]. Loss of ovarian hormones have been theorized as the cause of cognitive complaints and SCD in women undergoing menopause [30]. However, declines in ovarian hormones do not explain the rebound of memory that prior large longitudinal studies report among post-menopausal women [31, 32] and that is consistent with our findings. Whether memory problems during the menopausal transition are due to menopausal symptoms, including vasomotor symptoms, sleep, anxiety, and depression or due to other factors warrants further research [33].

Regarding sex differences in modifiable factors for SCD, we, along with a prior study using data from the 2011 BRFSS SCD module [34] found no appreciable differences between men and women in prevalence or risk factors for SCD. Findings on sex differences in AD and related dementia risk is also limited; and at present equivocal [12, 35]. For example, research from a subset of the Framingham Heart Study (1975–2009) found lifetime risk of Alzheimer’s disease and related dementia at age 45 to be 1 in 5 in women compared to 1 in 10 in men [12], while research from the Rochester Epidemiology Project (1985–1989) found no difference in risk between men and women [35]. Differences in these two studies highlight the need to consider study population characteristics when assessing sex-specific Alzheimer’s disease and related dementia risk. Furthermore, even if incidence or prevalence of SCD or dementia is equal in men and women in any given population, as we found in the BRFSS data, risk factors for SCD and dementia may still differ [35]. Sex differences for AD and dementia have been found for depression, sleep apnea, low education, marital status, and reproductive events unique to each sex (such as pregnancy and menopause for women and androgen-deprivation therapy for men) [23, 35]. Additionally, emerging research is indicating the importance of mid-life physical activity in reducing dementia risk [8], and sex differences in activity patterns and cognition [36, 37]. Clearly, despite our finding of no appreciable differences in contributing factors for SCD between men and women, more research is warranted assessing other risk factors that could not be assessed in this analysis.

## Strengths and limitations

Our study addressed prior gaps in the literature, including being one of the first studies to use recent data from a large population-based survey to assess PAFs of modifiable factors for SCD, while considering correlation between risk factors. This differs from other studies, which have focused on Alzheimer’s disease or all-cause dementia, versus SCD, or that have relied on meta-analyses for adjusted RR estimates [8–10], or which rely on unique cohorts from other countries which may not be generalizable to the US population [24, 25]. Despite our unique contribution, our study had several limitations. First, information for SCD and risk factors was based on self-reported data, which is susceptible to measurement error, especially since people experiencing cognitive decline are more likely to have anosognosia or be unaware of their deficits [38]. While anosognosia is associated with disease severity [39] and consequently individuals with SCD are less likely to be susceptible to anosognosia than individuals with Alzheimer’s disease or other dementia, we realize that anxiety around cognitive performance may still result in differential misclassification of the outcome or differential selection related to participants with SCD skipping over the cognitive questions. Additionally, individuals with severe SCD are most likely excluded since BRFSS respondents who complete the survey are deemed by themselves or another household member to be mentally fit to respond to the survey [40]. While the BRFSS finalized 2015 Cognitive Decline module includes only one question to determine SCD (with an additional five questions to assess whether SCD affects respondent’s functioning), further research using multi-item measures of SCD [41] would help differentiate how modifiable factors may impact various SCD subtypes (e.g., amnestic versus non-amnestic SCD or single-domain versus multiple-domain SCD) [42].

While self-report of cognitive decline and risk factors for cognitive decline have their limitations, they also have their strengths. For example, we used BRFSS questions that were better aligned with perceived social isolation rather than risk factors for social isolation, such as number of people living in household. This is important since perceived social isolation is usually more qualitative than quantitative in nature [43]. Another limitation of this study is the cross-sectional nature of the BRFSS survey, limiting ability to distinguish cause and effect. Only limited education, as defined, would precede SCD. To best assess risk, prospective cohort studies following young adults longitudinally, capturing modifiable factors over time and subsequent SCD are warranted. Longitudinal studies would also allow better capture of time-varying confounders or modifiers that may impact risk factors and SCD, such as personality factors or depression [44, 45]. Finally, we had incomplete data on hypertension, deafness, and most notably social isolation; and failed to include newly reported modifiable risk factors, including heavy alcohol consumption, traumatic brain injury, or air pollution, as reported in the 2020 by the *Lancet* Commission [8, 9]. Other potentially modifiable factors known to differ between men and women in relation to AD or related dementia risk, inclusive of cardiometabolic and reproductive health factors, also warrant further research.

## Conclusion

In summary, in this large nationally representative US sample of community-dwelling adults, we found that roughly 1 in 10 reported SCD with nearly 40% attributable to modifiable factors, including social isolation, depression, and hypertension, which explained nearly three-quarters of the attributable fraction. Further research, ideally in longitudinal cohorts assessing time-varying risk factors and SCD from early to late adulthood, is needed before definitive conclusions can be made. However, given recent findings from the SPRINT MIND trial showing that intensive blood pressure control can significantly reduce the risk of mild cognitive impairment [46], our findings documenting the high attributable fraction of hypertension in relation to SCD in a community-based sample suggest that blood pressure control may mitigate risk of SCD in over 50% of the US population over age 45. Additionally, prior research suggesting the positive link between social relationships and cognitive functioning [47] or long-term selective serotonin reuptake inhibitor treatment for depression and delayed progression to dementia [48] suggest early intervention strategies may have a significant impact on reducing or at least delaying cognitive impairment within the US population. While our study indicated minimal difference in prevalence or risk factors for SCD between sexes, further research assessing reproductive and endocrinological health history in addition to biological factors that interact with sex-related factors that can be modified [13] should be conducted within population-based samples.

## Supplementary Information

**Additional file 1.** Method for calculation of population attributable fraction and communality.

**Additional file 2.** Probability of SCD by Sex and Age Group of U.S. adults aged 45 years, 2015–2018.

**Additional file 3.** Adjusted modifiable risk factors for subjective cognitive decline with interaction to income, in U.S. adults aged 45 years and older, 2015–2018.

**Additional file 4.** Adjusted modifiable risk factors for subjective cognitive decline in U.S. adults aged 45–59 years old, 2015–2018.

**Additional file 5.** Adjusted modifiable risk factors for subjective cognitive decline in U.S. adults aged 60-74 years old, 2015–2018.

**Additional file 6.** Adjusted modifiable risk factors for subjective cognitive decline in U.S. adults aged 75 years or older, 2015–2018.

## Data Availability

BFFSS is a publically available dataset overseen by the Centers for Disease Control and Prevention. Survey data and documentation are available at https://www.cdc.gov/brfss/data_documentation/index.htm.
