# Supplementary material for: Overall and sex-specific risk factors for subjective cognitive decline: findings from the 2015–2018 Behavioral Risk Factor Surveillance System Survey
Source: Biol Sex Differ. 2022 Apr 12;13:16. doi: 10.1186/s13293-022-00425-3 (PMC9004039; doi:10.1186/s13293-022-00425-3)
Supplement: Supplementary file 1 — Additional file 1. Method for calculation of population attributable fraction and communality. [file 13293_2022_425_MOESM1_ESM.docx]

**Additional file 1**

1. ${PAF}_{i}=\frac{P_{i} ({RR}_{i}-1)}{1+P_{i} ({RR}_{i} - 1)}$
2. $W_{i}= 1- {communality}_{i}$
3. $PAF_{overall}= 1-\prod_{i=1}^{n} (1- PAF_{i})$
4. $WPAF_{overall}= 1-\prod_{i=1}^{n} (1- W_{i}\cdot PAF_{i})$
5. $WPAF_{i}= \frac{PAF_{i}\cdot{WPAF}_{overall}}{\sum_{i=1}^{n} {PAF}_{i}}$

*i*, risk factor for subjective cognitive decline

*P*, prevalence of risk factor

*RR*, relative risk of subjective cognitive decline from a risk factor

*W*, weight

*(W)PAF*, (weighted) population attributable fraction
