# Supplementary figures and images for: Overall and sex-specific risk factors for subjective cognitive decline: findings from the 2015–2018 Behavioral Risk Factor Surveillance System Survey
Source: Biol Sex Differ. 2022 Apr 12;13:16. doi: 10.1186/s13293-022-00425-3 (PMC9004039; doi:10.1186/s13293-022-00425-3)

**Additional file 2:** Probability of SCD by Sex and Age Group of U.S. adults aged 45 years, 2015–2018

**
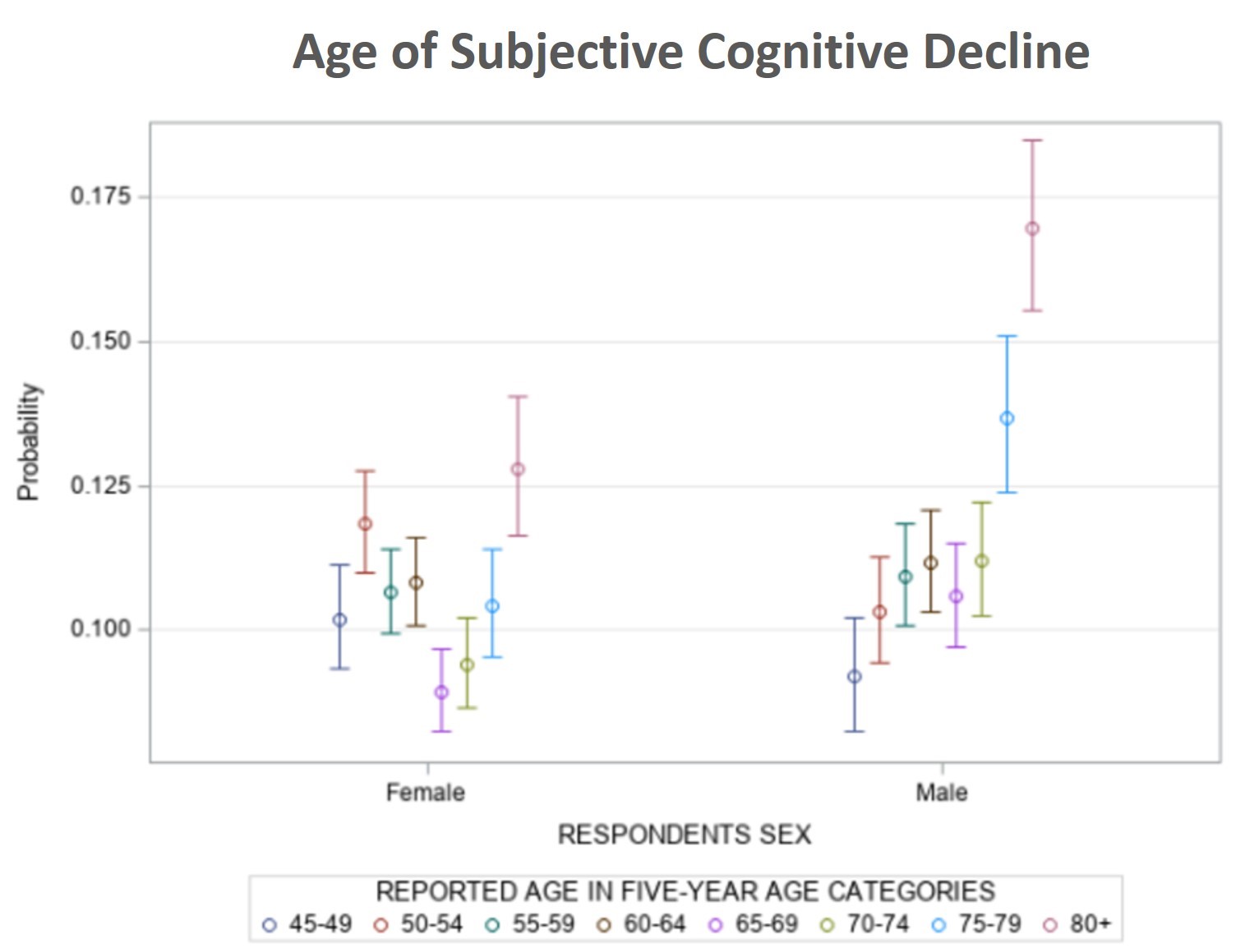
**

Supplement: Supplementary file 2 — Additional file 2. Probability of SCD by Sex and Age Group of U.S. adults aged 45 years, 2015–2018. [file 13293_2022_425_MOESM2_ESM.docx]
