# Supplementary material for: Overall and sex-specific risk factors for subjective cognitive decline: findings from the 2015–2018 Behavioral Risk Factor Surveillance System Survey
Source: Biol Sex Differ. 2022 Apr 12;13:16. doi: 10.1186/s13293-022-00425-3 (PMC9004039; doi:10.1186/s13293-022-00425-3)
Supplement: Supplementary file 3 — Additional file 3. Adjusted modifiable risk factors for subjective cognitive decline with interaction to income, in U.S. adults aged 45 years and older, 2015–2018. [file 13293_2022_425_MOESM3_ESM.docx]

**Additional file 3**. Adjusted modifiable risk factors for subjective cognitive decline with interaction to income, in U.S. adults aged 45 years and older, 2015–2018

| **Risk factor** | **Adj RR^1^ (95% CI^2^)** | **Prevalence (%)** | **Communality^3^ (%)** | **Adj PAF^4^ (%)** | **Weighted Adj PAF^5^ (%)** |
| --- | --- | --- | --- | --- | --- |
|  |  |  |  |  |  |
| **All Adults** | | | | | |
| Limited education*^a^ | 1.28 (1.04-1.52) | 4.78 | 66.19 | 1.32 | 0.44 |
| Deafness^b^ | 2.03 (1.83-2.22) | 9.66 | 39.81 | 9.02 | 3.03 |
| Social isolation*^c^ | 2.35 (2.07-2.63) | 52.38 | 70.97 | 41.37 | 13.88 |
| Depression^d^ | 3.10 (2.92-3.27) | 18.76 | 56.15 | 28.25 | 9.48 |
| Smoking^e^ | 1.19 (1.10-1.27) | 15.46 | 64.26 | 2.79 | 0.94 |
| Physical inactivity*^f^ | 1.31 (1.24-1.38) | 30.76 | 39.83 | 8.68 | 2.91 |
| Obesity*^g^ | 1.12 (1.06-1.18) | 32.97 | 59.06 | 3.93 | 1.32 |
| Hypertension^h^ | 1.28 (1.19-1.36) | 50.99 | 57.99 | 12.45 | 4.18 |
| Diabetes*^i^ | 1.29 (1.22-1.36) | 17.76 | 57.65 | 4.88 | 1.64 |
|  |  |  | **Overall^6^** | 73.18 | 37.81 |
|  |  |  |  |  |  |
| **Women** | | | | | |
| Limited education^a^ | 3.21 (2.96-3.45) | 4.49 | 58.99 | 9.03 | 3.14 |
| Deafness^b^ | 1.23 (0.88-1.57) | 7.46 | 48.90 | 1.67 | 0.58 |
| Social isolation*^c^ | 1.27 (1.16-1.38) | 55.70 | 69.20 | 13.09 | 4.55 |
| Depression^d^ | 2.06 (1.77-2.35) | 22.92 | 57.33 | 19.54 | 6.79 |
| Smoking^e^ | 1.36 (1.26-1.47) | 14.41 | 65.83 | 4.98 | 1.73 |
| Physical inactivity*^f^ | 1.28 (1.17-1.40) | 32.25 | 41.35 | 8.32 | 2.89 |
| Obesity*^g^ | 1.34 (1.24-1.44) | 32.08 | 64.84 | 9.87 | 3.43 |
| Hypertension^h^ | 1.14 (1.06-1.22) | 49.57 | 58.25 | 6.38 | 2.22 |
| Diabetes*^i^ | 2.37 (1.99-2.75) | 16.73 | 59.35 | 18.60 | 6.46 |
|  |  |  | **Overall^6^** | 62.57 | 31.79 |
|  |  |  |  |  |  |
| **Men** | | | | | |
| Limited education^a^ | 1.23 (0.88-1.57) | 5.11 | 26.67 | 1.15 | 0.44 |
| Deafness^b^ | 2.06 (1.77-2.35) | 12.22 | 20.30 | 11.46 | 4.39 |
| Social isolation*^c^ | 2.37 (1.99-2.75) | 47.68 | 71.96 | 39.44 | 15.10 |
| Depression^d^ | 3.21 (2.96-3.45) | 13.94 | 39.75 | 23.53 | 9.01 |
| Smoking^e^ | 1.28 (1.17-1.40) | 16.68 | 50.75 | 4.48 | 1.72 |
| Physical inactive*^f^ | 1.34 (1.24-1.44) | 29.03 | 35.75 | 9.01 | 3.45 |
| Obesity^g^ | 1.14 (1.06-1.22) | 33.93 | 48.34 | 4.46 | 1.71 |
| Hypertension^h^ | 1.27 (1.16-1.38) | 52.63 | 57.98 | 12.46 | 4.77 |
| Diabetes*^i^ | 1.36 (1.26-1.47 | 18.95 | 54.94 | 6.45 | 2.47 |
|  |  |  | **Overall^6^** | 72.44 | 43.04 |
| Data aggregated from U.S. Behavioral Risk Factor Surveillance System, 2015-2018; estimates were weighted and/or adjusted for complex survey design. Not all risk factors were available for every year of the survey. RR and PAF were adjusted (adj) for race, income, employment status, marital status, and veteran status. | | | | | |
| *Denotes significant interaction with income. | | | | | |
| ^1^Relative risk and ^2^confidence intervals for subjective cognitive decline. ^3^Total amount of variance a risk factor shares with the other factors. | | | | | |
| ^4^Population attributable fraction; proportion of all cases of subjective cognitive decline in the population that is attributable to a risk factor. | | | | | |
| ^5^PAF after accounting for communality. ^6^Combined PAF of all risk factors. ^3-6^Please see *Appendix* for formulas. | | | | | |
| ^a^Never attended school or discontinued after 8^th^ grade. | | | | | |
| ^b^Includes serious difficulty hearing. | | | | | |
| ^c^Never or rarely receive needed social and emotional support and/or have social activities significantly limited by arthritis. | | | | | |
| ^d^Ever told to have a depressive disorder. | | | | | |
| ^e^Currently smoke every day or some days and have smoked ≥ 100 cigarettes in lifetime. | | | | | |
| ^f^No physical activity or exercise during past 30 days other than regular job. ^g^Body mass index (BMI) ≥ 30.00 kg/m^2^. | | | | | |
| ^h^Ever told to have high blood pressure by a health professional, excluding during pregnancy or borderline high, pre-hypertension. | | | | | |
| ^i^Ever told to have diabetes, excluding gestational, borderline or pre-diabetes. | | | | | |
